# Wry-Neck

**Published:** 1889-02-02

**Authors:** 


### Within the Wards.

### WRY-NECK.

Wry-neck is not a very uncommon deformity. It is decidedly unsightly in children, and must be a source of real distress of mind to grown-up persons. It does not seem to be generally known that the cure of it by surgical operation is, as a rule, quite easy; so easy indeed that every wry-necked child or adult ought at least to be examined by a surgeon, and treated if he decides in favour of an operation. The case of a little girl reported from the Southport Infirmary will make clear to non-professional readers the simple history and value of an operation for wry-neck. The child was eight years old when seen by the surgeon, and had been wry-necked from birth. As she grew older the deformity increased very greatly. When taken to the infirmary the head was drawn down and turned much to the right; the mouth and eyelids were also drawn down, and gave an altered expression to the face, which was raised, and a spinal curvature had begun to show itself. Here was a series of deformities and disabilities of a very grave character, sufficient to make that little girl, if left alone, not only unlovely and singular, but unhappy and dissatisfied for the rest of her life. The infirmary surgeon decided upon an operation. It is not necessary to describe what he did. Professional readers will know, and lay readers would not understand. He did, however, operate by cutting, and did not tinker with collars and other mechanical appliances, which, in most serious cases, are as useless as physic would be. But the result is well worth describing. Immediately after the cutting of the faulty muscle, the head became suddenly straight. Then the wound healed, and afterwards massage was performed upon the neck and face, with a view to restoring the natural appearance of the mouth and eyes. A weight was frequently carried in the hand to draw the shoulder down. Finally a complete cure was effected. The head and face are now perfectly straight, and the spinal curvature has entirely disappeared. If, after reading an account of this kind, any parent or guardian who may be responsible for a wry-necked child should fail to entrust him to a competent surgeon, will not the child, on growing up, have reason to say that his parent or guardian behaved towards him in childhood with a negligence both reprehensible and criminal?

